# Predictors of Anxiety, Depression, and Stress in Long COVID: Systematic Review of Prevalence

**DOI:** 10.3390/ijerph22060867

**Published:** 2025-05-31

**Authors:** Daniel de Macêdo Rocha, Andrey Oeiras Pedroso, Mayra Gonçalves Menegueti, Renata Cristina de Campos Pereira Silveira, Laelson Rochelle Milanês Sousa, Elucir Gir, Renata Karina Reis

**Affiliations:** 1 Department of Nursing, Federal University of Mato Grosso do Sul, Coxim 79400-000, Brazil; 2 Ribeirão Preto College of Nursing, University of São Paulo, Ribeirão Preto 14040-902, Brazil; apedroso@usp.br (A.O.P.); mayramenegueti@usp.br (M.G.M.); recris@eerp.usp.br (R.C.d.C.P.S.); egir@eerp.usp.br (E.G.); rkreis@eerp.usp.br (R.K.R.); 3 Nursing Course, State University of Maranhão, Coroatá 65665-000, Brazil; laelsonsousa@professor.uema.br

**Keywords:** acute post-COVID-19 syndrome, anxiety, depression, stress, prevalence, risk factor

## Abstract

Anxiety, depression, and stress are prevalent psychosocial manifestations in Long COVID, and understanding their global impact can guide safe, effective, and evidence-based interventions. This study reviewed the literature to analyze the prevalence indicators and predictors of anxiety, depression, or stress experienced by adults and older adults with Long COVID. This systematic prevalence review was conducted using the databases MEDLINE via PubMed^®^, CINAHL-EBSCO, Web of Science, Scopus, EMBASE, LILACS, and BDENF. Observational studies that assessed anxiety, depression, or perceived stress in adults and older adults with Long COVID were included, with no restrictions on time or language. Two reviewers independently conducted the selection process. Full texts were analyzed for their eligibility potential. Methodological quality was assessed using the JBI Critical Appraisal Checklist for Studies. Ten observational studies with moderate methodological quality were included. Anxiety and depression were the most prevalent psychosocial symptoms in Long COVID, reported in mild, moderate, and severe cases of COVID-19 infection. Prevalence rates reached up to 47.8% for anxiety, 37.3% for depression, and 23% for stress. The combined analysis revealed a pooled prevalence of 15.3% (95% CI: 10.8% to 20.2%). Being female, having pre-existing mental disorders or associated clinical comorbidities, experiencing severe infection in the acute phase, and receiving intensive care were predictors of greater mental burden. The experience of anxiety, depression, and stress in prolonged COVID-19 was reported in countries with different income levels and was disproportionately experienced, especially by women and individuals with associated clinical conditions or psychopathological comorbidities.

## 1. Introduction

Long COVID constitutes a complex, multidimensional, progressive, and impactful syndrome, representing a global challenge in countries with different income levels. Despite variations in definitions, the World Health Organization defines Long COVID as the development of residual symptoms typically three months after the initial COVID-19 infection, persisting for at least two months and not explained by an alternative diagnosis. Previous studies have documented physical and neuropsychiatric symptoms that persist up to 12 weeks after the onset of the acute phase of COVID-19 [1,2].

In 2022, Long COVID affected the quality of life of over 65 million individuals who had contracted COVID-19 [3]. Incidence estimates vary from 10% to 30% among non-hospitalized cases and may reach up to 70% among patients who required hospitalization for infection treatment [4,5]. Even among vaccinated individuals, significant clinical signs of the syndrome have been reported [6,7]. In Brazil, positive screening for mental health events related to Long COVID has been identified among adults and older adults across all regions of the country, in diverse sociodemographic, clinical, and health contexts [8].

Although Long COVID encompasses a wide clinical and laboratory spectrum with a continuous, severe, and recurrent impact, the high mental health burden is widely referenced [9,10]. Events related to anxiety, depression, and stress may persist and lead to severe impairments in functional capacity and absenteeism indicators, in addition to contributing to loss of productivity, reduced quality of life, and increased suicide risk. While the acute phase of COVID-19 has been well characterized, understanding the mental health impairments of Long COVID has not yet been systematically consolidated. Moreover, its predictors remain poorly explored, constituting important gaps in scientific knowledge [11,12,13].

Significant efforts have been made to describe, study, and understand the clinical manifestations of the syndrome and its repercussions on physical and mental health, considering that Long COVID overloads health systems and presents significant morbidity. Furthermore, understanding mental health outcomes is essential for the development, direction, and effectiveness of comprehensive, sustainable, and evidence-based care strategies and public policies [14].

In this study, the following research hypothesis was considered: Sociodemographic, clinical, and therapeutic conditions affect the prevalence of anxiety, stress, or depression in Long COVID. Therefore, our objective was to analyze the prevalence indicators and predictors of anxiety, depression, or stress experienced by adults and older adults with Long COVID.

## 2. Materials and Methods

The methods adopted for the systematic review of prevalence were based on the guidelines of the Preferred Reporting Items for Systematic Reviews and Meta-Analyses (PRISMA) [15,16], and the protocol was registered on the Open Science platform (DOI: 10.17605/OSF.IO/3AX4Z) [17].

The research question “What is the prevalence and predictors of anxiety, depression and/or stress perceived by adults and older adults with Long COVID?” was based on the PICo strategy. Under these conditions, the Population (P) considered for this study was adults and older adults; the phenomenon of Interest (I), anxiety, depression, and/or perceived stress; and the research Context (Co), Long COVID.

The electronic search was conducted on 27 October 2024, in the following databases: MEDLINE via PubMed^®^, CINAHL-EBSCO, Web of Science™, Scopus, EMBASE, LILACS, and BDENF via the Virtual Health Library (VHL). An additional search was carried out on Google Scholar, where the 100 most relevant studies were reviewed. We selected the controlled and uncontrolled descriptors indexed in the Medical Subject Headings (MeSH), Health Sciences Descriptors (DeCS), and Embase Thesaurus (Emtree Terms) vocabularies. Search operationalization was adapted for each database using the Boolean operators OR and AND (Table 1).

This review included observational studies that assessed anxiety, depression, or stress perceived by adults and older adults who experienced the psychosocial repercussions of COVID-19 infection on a prolonged and recurrent basis. For inclusion, the following terminologies were considered:

- Adults and older adults: Person who has reached full growth or maturity, aged 19 years or older [18].
- Anxiety: The organism’s adaptive response to a signal of danger or threat, characterized by physiological, behavioral, and cognitive conditions. It comprises a pathological situation where the level of activation or duration is disproportionate to the situation experienced [19,20].
- Depression: Change in mood and state of mental disturbance manifested by sadness, loss of interest and pleasure, feelings of guilt, low self-esteem, and changes in sleep [19,20].
- Stress: Situation of acute or chronic tension that produces a change in physical behavior and emotional status [21].
- Long COVID: Clinical syndrome expressed by persistent, recurrent, and prolonged physical, cognitive, and/or neuropsychiatric symptoms after the acute phase of COVID-19 infection, without alternative diagnosis or associated condition [1,2].

No limitations regarding language or period of publication were defined in the selection process. Exclusion was based on the following criteria: (1) duplicate records across the consulted databases; (2) assessment of anxiety, stress, or depression in pediatric populations; (3) investigation of mental burden during the acute phase of COVID-19 infection; (4) case series, technical reports, editorials, scientific abstracts, reviews, theses, books, or master’s dissertations.

The identified studies were exported to EndNote Basic Manager to remove duplicates. Subsequently, all references were imported into Rayyan platform, a manager that allows blind selection among reviewers. Subsequently, two independent reviewers assessed the titles and abstracts to determine the potential inclusion of studies identified in the selected databases. For studies deemed relevant, the full texts were reviewed. Disagreements between reviewers at any stage of the review were resolved by a third reviewer. Inter-rater agreement was estimated using the Kappa coefficient (0.92).

Data extraction was also conducted by pairs independently and blindly. We used a form proposed by the JBI developed and applied to analyze demographic, geographic, social, and health risk factors. The variables of interest were expressed by research setting, conditions and populations investigated, methodological design, sample composition, prevalence estimates, predictors, determinants, and factors associated with the outcomes of interest [17].

To assess the methodological quality, we used the JBI Critical Appraisal Checklist for Studies. It is a validated form composed of a structured checklist for measuring the attributes necessary for prevalence studies [17].

The analysis and synthesis of results were carried out descriptively. It is noteworthy that the evidence presented in this study is of a secondary nature. Therefore, no approval by a Research Ethics Committee was necessary.

## 3. Results

**Figure 1:**
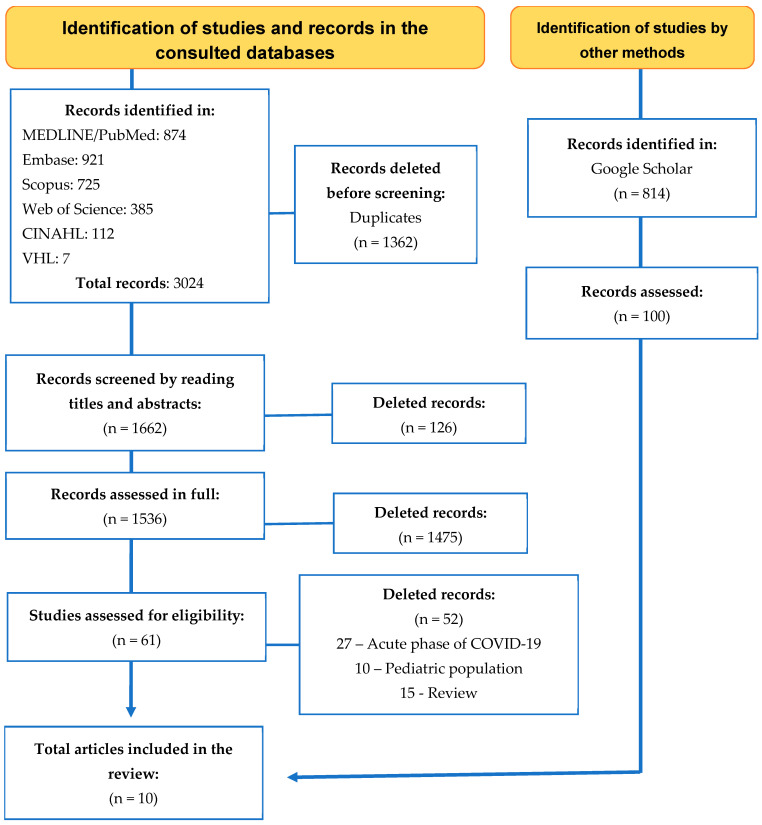
Selection and eligibility process and inclusion of studies in the review on the prevalence and predictors for anxiety, depression, and stress in Long COVID. Source: Preferred Reporting Items for Systematic Reviews and Meta-Analysis.

Search operationalization in the databases of interest favored the identification of 3024 records, of which 1362 were removed due to duplication. The record was maintained on specific health bases, followed by multidisciplinary ones, resulting in the assessment of 1662 studies regarding their potential for inclusion. Of these, 61 were selected for full reading and 10 that met the eligibility criteria were considered for sample composition. Figure 1 shows the identification, screening, selection, and inclusion path.

The analyzed investigations were conducted and published between 2021 and 2022, with sample sizes ranging from 100 to 273,618 adults and older adults diagnosed with COVID-19 infection. Long COVID emerged as a significant phenomenon among the studied participants, and mental health outcomes have attracted considerable interest in both scientific and healthcare fields.

The comparative analysis of the studies reveals both points of convergence and methodological and contextual divergences. Despite variations in study design, all investigations adopted observational methodologies, which are appropriate for examining persistent symptoms and their associated factors in populations previously infected with SARS-CoV-2.

A striking similarity among the studies is the identification of risk factors and determinants for psychosocial symptoms in Long COVID. Female sex was identified as one of the main predictors in several studies, suggesting a higher mental health burden among women. Additionally, the presence of clinical comorbidities, a prior psychiatric history, and greater severity of the acute phase of infection were also associated with an increased risk of developing anxiety and depression.

However, important differences were observed regarding the sample sizes and participant profiles. While some studies included large population-based cohorts, others were based on smaller, more restricted samples. The average age of participants also varied widely across studies. Another factor contributing to heterogeneity was the timing of symptom assessment after infection. Symptom monitoring began as early as 1 month after hospital treatment and extended up to 12 months after symptom onset. Studies that assessed patients at earlier stages tended to report higher prevalence rates of anxiety, stress, and depression symptoms.

The prevalence of anxiety varied significantly across studies, ranging from 7.1% to as high as 47.8%. For depression, reported rates ranged from 2.8% to 37.3%. Combined stress prevalence also appeared at different levels. Studies that simultaneously investigated multiple symptoms identified high rates of co-occurrence between anxiety, depression, and stress.

Geographical diversity across the studies is also notable. Surveys conducted in high-income countries such as the United States, the United Kingdom, and Italy focused on specific psychopathological symptoms using hospital-based samples. In contrast, the study conducted in Brazil addressed socioeconomic variables and social vulnerability, such as sex, lifestyle habits, and chronic diseases.

The characterization and synthesis of the results are presented in Table 2, which organizes the included studies according to theoretical frameworks, objectives, methodological design, and research outcomes. It also describes the prevalence and predictors of anxiety, depression, or stress among individuals who experienced Long COVID across the different contexts analyzed.

The methodological quality of the evaluated studies was mostly moderate to good, with all studies providing a clear description of inclusion criteria, subjects, and study settings, and using valid and reliable methods to measure outcomes such as anxiety, depression, and stress. However, most of the studies did not identify or adopt effective strategies to address confounding factors, such as comorbidities and pre-existing mental health conditions. Although the outcomes were measured appropriately, the statistical analysis was not always suitable for dealing with data variability and adjusting for confounding variables (Table 3).

**Figure 2:**
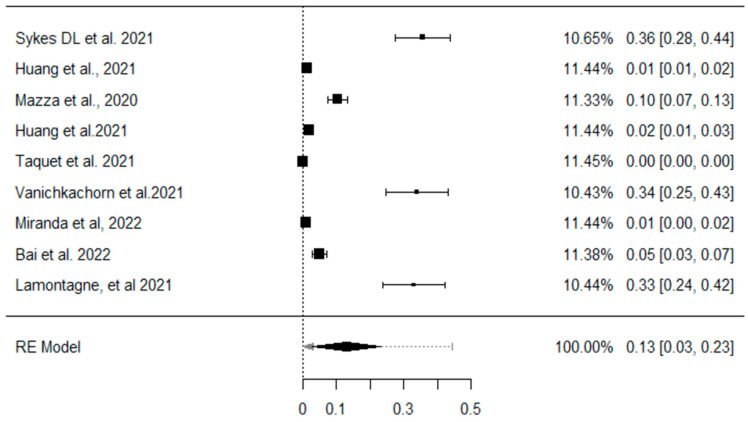
Prevalence of anxiety, depression, or stress. Forest graph of the meta-analyses of studies included on the prevalence of anxiety, depression, and stress in Long COVID. Note: Graph generated by the RevMan statistical program. CI = Confidence Interval. Skyes DL et al., 2021 is [21], Huang et al., 2021 is [22], Mazza et a., 2020 is [23], huang et al., 2021 is [24], Taquet et al., 2021 is [25], Vanichkachorn et al., 2021 is [26], miranda et al., 2022 [27], Bai et al., 2022 is [28], Lamontagne et al., 2021 is [29].

Figure 2 shows the overall prevalence of anxiety, depression, and/or stress in patients who experienced Long COVID. The individual estimates varied considerably across studies, reflecting heterogeneity in the populations assessed and in the data collection methods. The weight assigned to each study varied, possibly reflecting differences in sample size and the precision of the estimates. The pooled analysis revealed a combined prevalence of 15.3% (95% CI: 10.8% to 20.2%) for symptoms of anxiety, stress, and depression.

## 4. Discussion

The results of this systematic review confirm that the psychosocial repercussions of Long COVID represent a global challenge, given the significant prevalence among adult and elderly populations, as well as the prolonged duration and high potential for recurrence. Reports on the syndrome are still recent, and this review demonstrated both the prevalence indicators and the predictors for the development or worsening of anxiety, depression, and perceived stress following a confirmed COVID-19 infection.

Efforts to elucidate the psychiatric sequels of COVID-19 are increasing and were expressed in this study by the number of studies developed in different contexts and levels of healthcare. Sample composition, involving significant samples, mostly represented by adults undergoing different treatment modalities, stands out.

Long COVID continues to generate discussions across scientific, political, and healthcare domains, and its occurrence has been reported across all stages of acute COVID-19 infection. Mental health problems were observed both in patients who experienced moderate and severe cases of COVID-19, as well as in individuals with mild manifestations of the infection [22,24,27]. The development of psychosocial repercussions occurred after four weeks from the acute phase of the disease and was not associated with an alternative diagnosis [29].

Although it is plausible to consider that individuals with psychiatric diagnoses of anxiety, mood disorders, or stress may have a history of pre-existing chronic conditions, the studies included in this review predominantly point to the development or worsening of these symptoms following the acute phase of COVID-19 infection. Several investigations employed rigorous temporal criteria, assessing participants weeks or months after the resolution of the acute phase, which supports the inference that such psychosocial manifestations are temporally associated with Long COVID.

Furthermore, even in cases where there was a prior history of psychiatric disorders, symptoms of anxiety, depression, or stress were frequently reported as new, recurrent, or intensified in the post-infection period. Studies reinforce this perspective by identifying a significant increase in mental health burden among patients with no previous history of mental disorders, suggesting that COVID-19 may act as a triggering or amplifying factor for these conditions [23,28].

The lasting symptom burden and impact of COVID-19 on patients were examined in one-, three-, six-, and twelve-month cohorts [23,24,25]. A longitudinal study demonstrated a prolonged burden of anxiety and depression as well as clinical worsening when comparing indicators at a 6-month interval [24].

Anxiety and depression comprised the most investigated outcomes [21,22,23,24,25,26,27,28,29,30]. In the literature, they are considered universal, complex, multidimensional, predictable phenomena with high mortality rates. These are conditions prevalent in the general population that can negatively impact different dimensions of quality of life and overall health [25,26,27].

The prevalence rates identified varied, which were significant and recorded in low-, middle-, and high-income countries. The highest rates of anxiety and depression occurred in patients admitted to hospital in England, although no influence was found on the level of care required [21]. A longitudinal follow-up carried out in Brazil with adults and older adults for up to 14 months showed the lowest prevalence rates: 7.1% for anxiety and 2.8% for depression [27].

Perceived stress has been investigated in Canada and the USA. A cohort that assessed the level of mood and cognitive functioning demonstrated that 23% of cases reported the presence of the change in people who recovered from the infection for a period of one to four months [29]. Post-traumatic stress disorder was also assessed, with a prevalence of up to 31.0% in Italy [28]. The highest coefficients occurred in ICU survivors and were evident according to the length of assessment (3 to 12 months).

The associations of these outcomes with loss of productivity, reduced functional capacity, and risk of suicide stand out. Furthermore, the chronic course of these disorders can contribute to reduced self-esteem and subjective well-being, in addition to increasing vulnerability to mental distress [21,22,27,29].

The higher occurrence of anxiety, depression, and stress related to Long COVID among women was reported in most studies, demonstrating that this demographic factor was an important predictor among adults and the elderly. Other epidemiological projections confirm this trend by showing a disproportionate distribution of the mental health burden between sexes [24,28]. These differences may be explained by a combination of biological, hormonal, social, cultural, and psychological factors. An exacerbated immune response to infections, hormonal fluctuations throughout life, the multiple social roles women perform, and their longer life expectancy may contribute to their greater vulnerability to experiencing Long COVID [15].

Another important predictor for anxiety, depression, and stress among adults and the elderly with Long COVID was the clinical severity of the infection during the acute phase. The included studies demonstrated that these events can be identified across all clinical presentations of the disease. Nevertheless, the greatest mental health burden was experienced by patients with severe cases, those who required intensive care, and those who needed ventilatory support to sustain life [22,24].

A profound impact on mental health was also reported among individuals of advanced age or those with associated clinical comorbidities. Pre-existing conditions such as obesity, diabetes mellitus, and systemic arterial hypertension may lead to severe clinical presentations and the need for intensive care. Moreover, COVID-19 mortality indicators are concentrated within these population groups. Being part of a high-risk group for severe infection or presenting clinical characteristics frequently associated with fatal outcomes contributes to the higher prevalence of anxiety, depression, and stress in Long COVID [27,28,30].

A pre-existing psychiatric diagnosis was also considered a risk factor. A history of previous disorders, especially anxiety and depression, reinforces the chronic course of these conditions and the need for continuous assessment for psychological stabilization and therapeutic maintenance. Despite this, outpatient care, a type of specialized care, was suspended with the structuring of the proposed measures for social distancing and isolation. This condition may have impacted the recurrence of crises, and the development or intensification of identified emotional reactions [23,24,25,26,27,28,29,30].

The main limitation of this study lies in the methodological, conceptual, contextual, geographical, and population-based variations among the evaluated studies. Although it estimates the prevalence and predictors of anxiety, depression, and stress in Long COVID, the definitions of Long COVID varied across studies, particularly regarding the temporal framework. Additionally, the investigation of outcomes related to prior psychiatric treatment and the use of psychotropic medications remains limited in the included studies. Underreporting of mental health indicators within this population group may represent another limiting factor, potentially affecting the accuracy of the identified outcomes. A final limitation of most of the included studies is the lack of control groups, which makes it difficult to distinguish between psychological symptoms specifically attributable to Long COVID-19 and those resulting from broader contextual factors. This limits causal interpretation and may lead to overestimation of the impact of Long COVID-19 on mental health. Future studies should include appropriate control groups to better isolate these effects.

## 5. Conclusions

The reviewed studies converge in identifying anxiety and depression as prevalent psychosocial manifestations of Long COVID, particularly among women, individuals with pre-existing health conditions, and those who experienced severe forms of the infection. However, differences in methodologies, contexts, and study populations suggest that the experience of mental distress in Long COVID is multifactorial, requiring intervention strategies tailored to the clinical and sociodemographic specificities of each population. The variability in findings also underscores the importance of long-term investigations and prospective study designs to assess the progression of symptoms over time, especially in populations facing diverse social and health contexts. Understanding the long-term psychosocial repercussions of COVID-19 can guide care planning and support the development of effective, sustainable, and evidence-based public policies.

## Figures and Tables

**Table 1:** Search strategy after consulting the databases of interest.

Database	Search Strategy
MEDLINE	((((((((((((“Post-acute COVID-19 syndrome”) OR (“Long-COVID”)) OR (“Post-COVID-19 syndrome”)) OR (“Long Haul Syndrome COVID-19”)) OR (“long-haul COVID”)) OR (“post-acute COVID syndrome”)) OR (“persistent COVID-19”)) OR (“long COVID”)) OR (“long haul COVID”)) OR (“chronic COVID syndrome”))) OR (“COVID survivor”)) AND ((((((Anxiety) OR (Anxiousness)) OR (Depression)) OR (“Depressive Symptoms”)) OR (((((“Stress Disorders, Traumatic”) OR (“Stress Disorders, Post-Traumatic”)) OR (“Traumatic Stress Disorder”)) OR (“Stress, Psychological”)) OR (“Psychological Stress”))))
Web of Science	(ALL = (“Post-acute COVID-19 syndrome”) OR ALL = (“Long-COVID”) OR ALL = (“Post-COVID-19 syndrome”) OR ALL = (“Long Haul Syndrome COVID-19”) OR ALL = (“long-haul COVID”) OR ALL = (“post-acute COVID syndrome”) OR ALL = (“persistent COVID-19”) OR ALL = (“long COVID”) OR ALL = (“long haul COVID”) OR ALL = (“chronic COVID syndrome”) OR ALL = (“COVID survivor”)) AND (ALL = (Anxiety) OR ALL = (Anxiousness) OR ALL = (Depression) OR ALL = (“Depressive Symptoms”) OR ALL = (“Stress Disorders, Traumatic”) OR ALL = (“Stress Disorders, Post-Traumatic”) OR ALL = (“Traumatic Stress Disorder”) OR ALL = (“Stress, Psychological”) OR ALL = (“Psychological Stress”))
Scopus	((TITLE-ABS-KEY (“Post-acute COVID-19 syndrome”) OR TITLE-ABS-KEY (“Long-COVID”) OR TITLE-ABS-KEY (“Post-COVID-19 syndrome”) OR TITLE-ABS-KEY (“Long Haul Syndrome COVID-19”) OR TITLE-ABS-KEY (“long-haul COVID”) OR TITLE-ABS-KEY (“post-acute COVID syndrome”) OR TITLE-ABS-KEY (“persistent COVID-19”) OR TITLE-ABS-KEY (“long COVID”) OR TITLE-ABS-KEY (“long haul COVID”) OR TITLE-ABS-KEY (“chronic COVID syndrome”) OR TITLE-ABS-KEY (“COVID survivor”))) AND ((TITLE-ABS-KEY (anxiety) OR TITLE-ABS-KEY (anxiousness) OR TITLE-ABS-KEY (depression) OR TITLE-ABS-KEY (“Depressive Symptoms”) OR TITLE-ABS-KEY (“Stress Disorders, Traumatic”) OR TITLE-ABS-KEY (“Stress Disorders, Post-Traumatic”) OR TITLE-ABS-KEY (“Traumatic Stress Disorder”) OR TITLE-ABS-KEY (“Stress, Psychological”) OR TITLE-ABS-KEY (“Psychological Stress”)))
Embase	(‘post-acute COVID-19 syndrome’ OR ‘long-COVID’ OR ‘long COVID’ OR ‘post-COVID-19 syndrome’ OR ‘long haul syndrome COVID-19’ OR ‘long-haul COVID’ OR ‘post-acute COVID syndrome’ OR ‘persistent COVID-19’ OR ‘long haul COVID’ OR ‘chronic COVID syndrome’ OR ‘COVID survivor’) AND (anxiety OR ‘anxiety disorder’ OR anxiousness OR depression OR ‘depressive symptoms’ OR ‘posttraumatic stress disorder’ OR ‘stress disorders, traumatic’ OR ‘stress disorders, post-traumatic’ OR ‘traumatic stress disorder’ OR ‘mental stress’ OR ‘stress, psychological’ OR ‘psychological stress’)
LILACS, BDENF and IBECS via VHL	((“Síndrome pós-COVID-19”) OR (“Síndrome pós-aguda de COVID-19”) OR (“COVID longa”) OR (“Síndrome de longo curso COVID-19”) OR (“COVID-19 persistente”) OR (“COVID de longa duração”) OR (“síndrome de COVID crônica”) OR (“sobrevivente de COVID”) OR (“Post-Acute COVID-19 Syndrome”) OR (“Long-COVID”) OR (“Post-COVID-19 syndrome”) OR (“Long Haul Syndrome COVID-19”) OR (“long-haul COVID”) OR (“post-acute COVID syndrome”) OR (“persistent COVID-19”) OR (“long COVID”) OR (“long haul COVID”) OR (“chronic COVID syndrome”) OR (“COVID survivor”)) AND ((mh:(Ansiedade)) OR (Ansiedade) OR (Angústia) OR (mh:(Depressão)) OR (Depressão) OR (“Sintomas Depressivos”) OR (mh: (“Estresse Psicológico”)) OR (“Estresse Psicológico”) OR (Estresse) OR (Anxiety) OR (Anxiousness) OR (Depression) OR (“Depressive Symptoms”) OR (“Stress Disorders, Post-Traumatic”) OR (“Stress Disorders, Traumatic”) OR (“Stress Disorders, Post-Traumatic”) OR (“Traumatic Stress Disorder”) OR (“Stress, Psychological”) OR (“Stress, Psychological”) OR (“Psychological Stress”))

**Table 2:** Characterization and synthesis of the studies included in the systematic review.

Author, Year, Periodical	Objective	Country	Design and Sample	Assessment Time Assessment Tool	Outcome (Prevalence) and Predictor
Sykes DL et al. [21]. 2021 Lung	Report the lasting burden of symptoms in patients admitted with COVID-19	England	Observational N = 134 Male: 65.7% Mean age: 59.6 Treated in wards: 80% Intensive Care Unit (ICU): 20%	113 days after hospital discharge Standardized Assessment Form and 5-level EuroQol-5 Dimension (EQ-5D-5L)	Anxiety (47.8%) and depression (37.3%) Predictors: Being female There were no significant differences in symptom duration based on level of care, maximum oxygen, or respiratory support received
Huang et al. [22]. 2021 Lancet	Describe the long-term consequences in patients with COVID-19 who have been discharged from hospital	China	Cohort N = 1733 Male: 52% Mean age: 57.0	6 months after the onset of symptoms Self-reported symptoms questionnaire, EQ-5D-5L questionnaire, and EuroQol Visual Analogue Scale (EQ-VAS)	Anxiety or depression (23%) Predictors: Being female, disease severity, use of supplemental oxygen
Mazza et al. [23]. 2020 Brain Behav Immun.	Investigate the psychopathological impact on COVID-19 survivors	Italy	Cross-sectional N = 402 Male: 56.7% Mean age: 57.80	One month after hospital treatment Impact of Events Scale-Revised (IES-R), Posttraumatic Stress Disorder Checklist for DSM-5 (PCL-5), Zung Self-Rating Depression Scale (ZSDS), 13-item Beck’s Depression Inventory (BDI-13), and State-Trait Anxiety Inventory form Y (STAI-Y)	Anxiety (42%), depression (28%), and post-traumatic stress disorder (28%). Predictors: Being female, previous psychiatric diagnosis, home treatment
Huang et al. [24]. 2021 Lancet	Compare outcomes between 6 months and 12 months after symptom onset among hospital survivors with COVID-19	China	Cohort 1276 Median age 59.0 years (IQR 49.0–67.0) 681 (53%) were men	6 months and 12 months after symptom onset Modified British Medical Research Council (mMRC) score and HRQoL	6 months Anxiety or depression (23%) 12 months Anxiety or depression (26%) Predictors: Being female, infection severity
Taquet et al. [25]. 2021 PLoS Med	Estimate the incidence, occurrence, and evolution of COVID-19 6 months after diagnosis	USA	Retrospective cohort N = 273,618 Female: 55.6% Mean age: 46.30	6 months after COVID-19 diagnosis. TriNetX Analytics to analyze demographics, diagnoses, and measurements	Anxiety or depression (15.49%) Predictor: Young adults
Vanichkachorn et al. [26]. 2021 Mayo Clin Proc.	Describe the characteristics of prolonged symptoms after COVID-19 infection	USA	Cohort N = 100 Female: 68% Mean age: 45.40	93 days after infection Electronic Health Records, EQ-5D-5L, EuroQol Visual Analogue Scale	Depression or anxiety (34%)
Miranda et al. [27]. 2022 Trans R Soc Trop Med Hyg.	Analyze the profile and symptoms suggestive of Long COVID	Brazil	Longitudinal N = 646 Female: 53.9% Mean age: 50.26	Up to 14 months Questionnaire and electronic medical records	Anxiety (7.1%) and depression (2.8%). Predictors: Age, disease severity, presence of comorbidities—high blood pressure, diabetes, heart disease, cancer, chronic obstructive pulmonary disease, chronic kidney disease, smoking, alcoholism
Bai et al. [28]. 2022 Clin Microbiol Infect	Identify the predictors of Long COVID	Italy	Prospective cohort N = 377	44 days Hospital Anxiety and Depression Scale (HADS) and Impact of Event Scale–Revised (IES-R)	Anxiety (18.8%), depression (10.6%), post-traumatic stress disorder (31%) Predictors: Being female and advanced age
Lamontagne, et al. [29]. 2021 Brain Behavior I. Health	Analyze mood and cognitive functioning after COVID-19 infection	USA Canada	Cohort N = 100 Female: 58.0% Mean age: 30.80	1–4 months Beck Depression Inventory-II (BDI-II), the Snaith-Hamilton Pleasure Scale (SHAPS), and the Mood and Anxiety Symptom Questionnaire (MASQ)	Anxiety (33%), perceived stress (23%), and depression (17%). Predictor: Headache in the acute phase
Tarsitani et al. [30]. 2021 J Gen Intern Med.	Assess the prevalence and risk factors of post-traumatic stress disorder in patients hospitalized for COVID-19 infection	Italy	Cohort N = 115 Male: 54%	3 months PCL-5	Post-traumatic stress disorder (10.4%) Predictor: Being female, previous psychiatric diagnosis, obesity

**Table 3:** Quality of evidence of studies included on the prevalence and predictors for anxiety, depression, and stress in Long COVID.

Study/Assessment Criteria	Were the Criteria for Inclusion in the Sample Clearly Defined?	Were the Study Subjects and the Setting Described in Detail?	Was the Exposure Measured in a Valid and Reliable Way?	Were Objective, Standard Criteria Used to Measure the Condition?	Were Confounding Factors Identified?	Were Strategies to Deal with Confounding Factors Stated?	Were the Outcomes Measured in a Valid and Reliable Way?	Was Appropriate Statistical Analysis Used?	Total
Sykes DL et al., 2021 [21]	Y	Y	Y	Y	N	N	Y	Y	6/8
Huang et al., 2021 [22]	N	Y	Y	Y	N	N	Y	Y	5/8
Mazza et al., 2020 [23]	Y	Y	Y	Y	N	N	Y	Y	6/8
Huang et al., 2021 [24]	Y	Y	Y	Y	N	N	Y	Y	6/8
Taquet et al., 2021 [25]	Y	Y	Y	Y	N	N	Y	Y	6/8
Vanichkachorn et al., 2021 [26]	Y	Y	Y	Y	N	N	Y	Y	6/8
Miranda et al., 2022 [27]	Y	Y	Y	Y	N	N	Y	Y	6/8
Bai et al., 2022 [28]	Y	Y	Y	Y	N	N	Y	Y	6/8
Lamontagne, et al., 2021 [29]	Y	Y	Y	Y	N	N	Y	Y	6/8
Tarsitani et al., 2021 [30]	Y	Y	Y	Y	N	N	Y	Y	6/8

Legend: Y—Yes; N—Not.

## References

[1] Davis H.E., McCorkell L., Vogel J.M., Topol E.J. (2023). Long COVID: Major findings, mechanisms and recommendations. Nat. Rev. Microbiol..

[2] Koc H.C., Xiao J., Liu W., Li Y., Chen G. (2022). Long COVID and its Management. Int. J. Biol. Sci..

[3] Ballering A.V., van Zon S.K.R., Hartman T.C.O., Rosmalen J.G.M. (2022). Lifelines Corona Research Initiative. Persistence of somatic symptoms after COVID-19 in the Netherlands: An observational cohort study. Lancet.

[4] Bull-Otterson L. (2022). Post–COVID conditions among adult COVID-19 survivors aged 18–64 and ≥65 years—United States, March 2020–November 2021. MMWR Morb. Mortal. Wkly. Rep..

[5] Ceban F., Ling S., Lui L.M., Lee Y., Gill H., Teopiz K.M., Rodrigues N.B., Subramaniapillai M., Di Vincenzo J.D., Cao B. (2022). Fatigue and cognitive impairment in Post-COVID-19 Syndrome: A systematic review and meta-analysis. Brain Behav. Immun..

[6] Al-Aly Z., Bowe B., Xie Y. (2022). Long COVID after breakthrough SARS-CoV-2 infection. Nat. Med..

[7] Ayoubkhani D., Bosworth M.L., King S., Pouwels K.B., Glickman M., Nafilyan V., Zaccardi F., Khunti K., Alwan N.A., Walker A.S. (2022). Risk of Long COVID in People Infected With Severe Acute Respiratory Syndrome Coronavirus 2 After 2 Doses of a Coronavirus Disease 2019 Vaccine: Community-Based, Matched Cohort Study. Open Forum Infect. Dis..

[8] de Macêdo Rocha D., Pedroso A.O., Sousa L.R.M., Gir E., Reis R.K. (2025). Predictors for Anxiety and Stress in Long COVID: A Study in the Brazilian Population. Int. J. Environ. Res. Public Health.

[9] Hawes M.T., Szenczy A.K., Klein D.N., Hajcak G., Nelson B.D. (2022). Increases in depression and anxiety symptoms in adolescents and young adults during the COVID-19 pandemic. Psychol. Med..

[10] Pérez-Cano H.J., Moreno-Murguía M.B., Morales-López O., Crow-Buchanan O., English J.A., Lozano-Alcázar J., Somilleda-Ventura S.A. (2020). Anxiety, depression, and stress in response to the coronavirus disease-19 pandemic. Cir. Cir..

[11] Wang S., Quan L., Chavarro J.E., Slopen N., Kubzansky L.D., Koenen K.C., Kang J.H., Weisskopf M.G., Branch-Elliman W., Roberts A.L. (2022). Associations of Depression, Anxiety, Worry, Perceived Stress, and Loneliness Prior to Infection With Risk of Post-COVID-19 Conditions. JAMA Psychiatry.

[12] Lee H.-Y., Choi D., Lee J.J. (2022). Depression, anxiety, and stress in Korean general population during the COVID-19 pandemic. Epidemiol. Health.

[13] Sher L. (2020). The impact of the COVID-19 pandemic on suicide rates. QJM.

[14] Ceban F., Kulzhabayeva D., Rodrigues N.B., Di Vincenzo J.D., Gill H., Subramaniapillai M., Lui L.M., Cao B., Mansur R.B., Ho R.C. (2023). COVID-19 vaccination for the prevention and treatment of long COVID: A systematic review and meta-analysis. Brain Behav. Immun..

[15] Moher D., Liberati A., Tetzlaff J., Altman D.G., Prisma Group (2009). Preferred reporting items for systematic reviews and meta-analyses: The PRISMA statement. PLoS Med..

[16] Munn Z., Moola S., Lisy K., Riitano D., Tufanaru C., Aromataris E., Munn Z. (2020). Chapter 5: Systematic reviews of prevalence and incidence. JBI Manual for Evidence Synthesis.

[17] Rocha D.M., Sousa L.R.M., Silveira R.C.C.P., Gir E., Reis R.K. (2023). Anxiety, depression and stress in long COVID syndrome: A systematic review protocol. Cent. Open Sci..

[18] Molina F.O. (1996). Estresse no Cotidiano. Comércio e Representações.

[19] Roy D., Tripathy S., Kar S.K., Sharma N., Verma S.K., Kaushal V. (2020). Study of knowledge, attitude, anxiety & perceived mental healthcare need in Indian population during COVID-19 pandemic. Asian J. Psychiatry.

[20] Lei L., Huang X., Zhang S., Yang J., Yang L., Xu M. (2020). Comparison of Prevalence and Associated Factors of Anxiety and Depression Among People Affected by versus People Unaffected by Quarantine During the COVID-19 Epidemic in Southwestern China. Med. Sci. Monit..

[21] Sykes D.L., Holdsworth L., Jawad N., Gunasekera P., Morice A.H., Crooks M.G. (2021). Post-COVID-19 Symptom Burden: What is Long-COVID and How Should We Manage It?. Lung.

[22] Huang C., Huang L., Wang Y., Li X., Ren L., Gu X., Kang L., Guo L., Liu M., Zhou X. (2021). 6-month consequences of COVID-19 in patients discharged from hospital: A cohort study. Lancet.

[23] Mazza M.G., De Lorenzo R., Conte C., Poletti S., Vai B., Bollettini I., Melloni E.M.T., Furlan R., Ciceri F., Rovere-Querini P. (2020). Anxiety and depression in COVID-19 survivors: Role of inflammatory and clinical predictors. Brain Behav. Immun..

[24] Huang L., Yao Q., Gu X., Wang Q., Ren L., Wang Y., Hu P., Guo L., Liu M., Xu J. (2021). 1-Year outcomes in hospital survivors with COVID-19: A longitudinal cohort study. Lancet.

[25] Taquet M., Dercon Q., Luciano S., Geddes J.R., Husain M., Harrison P.J. (2021). Incidence, co-occurrence, and evolution of long-COVID features: A 6-month retrospective cohort study of 273,618 survivors of COVID-19. PLoS Med..

[26] Vanichkachorn G., Newcomb R., Cowl C.T., Murad M.H., Breeher L., Miller S., Trenary M., Neveau D., Higgins S. (2021). Post-COVID-19 Syndrome (Long Haul Syndrome): Description of a Multidisciplinary Clinic at Mayo Clinic and Characteristics of the Initial Patient Cohort. Mayo Clin. Proc..

[27] Miranda D.A.P.d., Gomes S.V.C., Filgueiras P.S., Corsini C.A., Almeida N.B.F., Silva R.A., Medeiros M.I.V.A.R.C., Vilela R.V.R., Fernandes G.R., Grenfell R.F.Q. (2022). Long COVID-19 syndrome: A 14-months longitudinal study during the two first epidemic peaks in Southeast Brazil. Trans. R. Soc. Trop. Med. Hyg..

[28] Bai F., Tomasoni D., Falcinella C., Barbanotti D., Castoldi R., Mulè G., Augello M., Mondatore D., Allegrini M., Cona A. (2022). Female gender is associated with long COVID syndrome: A prospective cohort study. Clin. Microbiol. Infect..

[29] Lamontagne S.J., Winters M.F., Pizzagalli D.A., Olmstead M.C. (2021). Post-acute sequelae of COVID-19: Evidence of mood & cognitive impairment. Brain Behav. Immun. Health.

[30] Tarsitani L., Vassalini P., Koukopoulos A., Borrazzo C., Alessi F., Di Nicolantonio C., Serra R., Alessandri F., Ceccarelli G., Mastroianni C.M. (2021). Post-traumatic Stress Disorder Among COVID-19 Survivors at 3-Month Follow-up After Hospital Discharge. J. Gen. Intern. Med..

